# Simplified Chinese version of hip and knee replacement expectations surveys in patients with osteoarthritis and ankylosing spondylitis: cross-cultural adaptation, validation and reliability

**DOI:** 10.1186/s12891-018-2129-0

**Published:** 2018-07-21

**Authors:** Chen Wang, Chen Zhang, De-Lin Liu, Wen-Wen Tong, Chong-Ru He, Xuan Huang, Wei-Dong Xu

**Affiliations:** Department of Orthopedics, Changhai Hospital, Second Military Medical University, No. 168, Changhai Road, Shanghai, 200433 China

**Keywords:** Preoperative expectation, Questionnaire, Simplified Chinese version, Hip and knee arthroplasty, Reliability, Validity

## Abstract

**Background:**

The Hospital for Special Surgery Hip Replacement Expectations Survey (HSS-THRES) and Knee Replacement Expectations Survey (HSS-TKRES) are widely used tools developed to assess patients’ preoperative expectations for total hip and knee arthroplasty. This study aimed to translate and adapt the HSS-THRES and HSS-TKRES into Chinese versions (SC-THRES/TKRES) and evaluate their psychometric properties in patients with osteoarthritis (OA) and ankylosing spondylitis (AS).

**Methods:**

Patients scheduled for total hip (104 hip OA and 51 AS) or knee replacements (101 knee OA) were recruited in this study. Confirmatory Factor Analysis (CFA) was used to evaluate structural validity. The internal consistency was assessed by the Cronbach’s α coefficient. The intraclass correlation coefficient (ICC) was used to assess test-retest reliability. The construct validity was analyzed by evaluating the correlations between SC-THRES/TKRES and the Expectation WOMAC. The correlations with the Expectation WOMAC were tested against our hypotheses. We additionally compared preoperative expectations of AS patients to those of hip OA patients.

**Results:**

The results of CFA for the SC-THRES and SC-TKRES demonstrated good fit. The results for the SC-THRES/TKRES revealed good test-retest reliability and good internal consistency (AS: ICC = 0.893, Cronbach’s α = 0.815; hip OA: ICC = 0.878, Cronbach’s α = 0.814; knee OA: ICC = 0.806, Cronbach’s α = 0.808). The correlations between the SC-THRES/TKRES and the Expectation WOMAC were moderate (0.541 for AS, 0.490 for hip OA and 0.465 for knee OA), which were consistent with the hypotheses.

**Conclusion:**

The SC-THRES/TKRES are reliable, valid for the evaluation of Chinese patients with OA and AS undergoing total hip and knee arthroplasty. The surveys can be used as part of preoperative assessments. Meanwhile, additional research is needed to replicate these findings and to assess the content validity in a larger sample.

**Electronic supplementary material:**

The online version of this article (10.1186/s12891-018-2129-0) contains supplementary material, which is available to authorized users.

## Background

Total joint arthroplasties (TJAs) have proven to be effective surgical interventions for patients with moderate to severe hip or knee disorders [1]. The number of TJA procedures has doubled over recent decades and is expected to increase over the coming two decades [2]. Total hip arthroplasty (THA) and total knee arthroplasty (TKA) are extremely effective in reducing pain and restoring function in patients with end-stage osteoarthritis (OA) and ankylosing spondylitis (AS) [3–5].

More recently, increasing emphasis is focused on patient satisfaction and patient-reported outcomes of the surgery [6, 7]. Previous researches have indicated that fulfillment of preoperative expectations was one of the most important factors affecting satisfaction and was a large contributor to functional outcomes [8–13]. Fulfilled expectations correlate with increased patient compliance with postoperative recommendations, follow-up care and monitoring [14–17]. Several studies have shown that compared with the actual ability after surgery, preoperative expectations were higher [11, 18]. There was a large discrepancy between what patients expected and what they truly achieved. Additionally, more disabled patients often have higher preoperative expectations than their surgeons [19, 20]. Unrealistically high expectations may weaken the doctor-patient trust and cause patients to be discouraged after surgery [21]. Nowadays, as a consequence of the complexity of the healthcare environment, the doctor-patient relationship is increasingly tensional in China [22, 23]. Patients requiring TJA often have overly optimistic preoperative expectations, and many of them do not trust their surgeons. Thus, it is of great significance to investigate patients’ expectations before surgery. Currently there are no standard methods to assess expectations based on patient-derived measures.

Recent surveys have been developed and validated to capture patients’ expectations before THA or TKA [24–27]. The Hospital for Special Surgery Total Hip Replacement Expectations Survey (HSS-THRES) [27] and the Hospital for Special Surgery Total Knee Replacement Expectations Survey (HSS-TKRES) [25] are patient-reported surveys assessing patients’ preoperative expectations successfully, which have also been cross-culturally adapted to Dutch [28], French [29] and German [30]. To our knowledge, the HSS-THRES and the HSS-TKRES are the first two questionnaires developed to assess preoperative expectations for patients undergoing TJA. However, a Chinese version has yet to be developed. In addition, no study has proven to be a reliable and valid assessment of the HSS-THRES for patients with AS undergoing THA.

Therefore, the aims of this study were (1) to translate the original version of the HSS-THRES and the HSS-TKRES into Chinese, and (2) to evaluate the psychometric properties of the translated surveys in patients with AS and OA. We additionally compared preoperative expectations of AS patients to those of OA patients undergoing THA.

## Methods

### Patients

All 270 patients on the waiting list for primary THA/TKA from the orthopedic department of our hospital between November 2015 and July 2017 were invited to participate in this study. The major criteria for inclusion were as follows: (1) native Chinese speakers aged at least 18 years, (2) able to complete the questionnaire independently, (3) OA patients fulfilled the criteria of the American College of Rheumatology [31] with operative indications and willing to receive THA/TKA for the first time. (4) AS patients with hip ankylosis fulfilled the modified New York criteria [32], with operative indications and willing to receive THA for the first time. Subjects with (1) symptomatic OA in other joints, (2) a history of lower limb or spine surgery, (3) severe systematic illnesses were excluded from the study. The sample size was consistent with the study recommended by Terwee et al. [33]. Detailed information was recorded, including gender, age, body mass index (BMI), educational level and living situation. All the participants signed informed consent and the study was approved by the ethics committee of our university (No.CHEC2017–163).

### Translation and cross-cultural adaptation

First of all, the developer of the original questionnaires was informed of the purposes of the study and gave consent to a simplified Chinese translation of the HSS-THRES and HSS-TKRES (Carol Mancuso, MD, Hospital for Special Surgery, New York, USA, personal communication).

Based on the previously published guidelines [34], we carried out the cross-cultural adaptation and translation in five stages.

#### Stage I - forward translation

Three bilingual translators (mother-tongue Chinese and fluent in English) translated the questionnaire into simplified Chinese independently. Two of them are orthopedic surgeons in our hospital; the other one is a professional translator without any medical background who is unaware of the purpose of this study.

#### Stage II – Synthesis

The first synthetic version of the questionnaire was obtained by discussion of the three translators in order to resolve the discrepancies.

#### Stage III - back translation

The back translation of the first synthesis was completed separately by three native English speakers fluent in Chinese, totally blind to the original English version, and with no medical background.

#### Stage IV - expert committee review

The composition of the expert committee included the six translators from stage I and III, a linguist, a methodologist and two experts in orthopedics. Then the committee reviewed all the translations to resolve any discrepancies and drafted the pre-final version of the questionnaire.

#### Stage V – Pretesting

Twenty patients (more details were shown in Additional file 1) who were ready to undergo THA/TKA in our hospital were recruited to fill in the pre-final version. The final simplified Chinese version (SC-THRES/TKRES) was generated after the committee had resolved all the problems recognized in the pre-testing.

### Instruments

#### Hospital for Special Surgery Total hip Replacement Expectations Survey (HSS-THRES)

The HSS-THRES is designed by Mancuso et al. to investigate patients’ expectations before hip arthroplasty [27]. It is a self-reported questionnaire composed of 18 items relating to pain, physical activity, social activity and psychological well-being. Patients were asked how much improvement or relief they expect for each item followed by THA. The response format used in the questionnaire was as follows: ‘back to normal or completely improvement, ‘a lot of improvement’, ‘a moderate amount of improvement’, ‘a little improvement’ or ‘this expectation does not apply to me or I do not have this expectation’. The total score ranging from 0 to 72 was adapted into a 100-point scale, with a higher score representing higher expectations. The original English version showed good reliability and construct validity [27, 35].

#### Hospital for Special Surgery Total Knee Replacement Expectations Survey (HSS-TKRES)

Similarly, the HSS-TKRES is a 19-item questionnaire developed by Mancuso et al. [25]. The response format was similar to the one used in HSS-THRES. The total score was normalized to values between 0 (no expectation) and 100 (highest expectation). The initial English-language survey showed good reliability and construct validity [25, 35].

### Expectation WOMAC

To determine construct validity, we compared the SC-THRES/TKRES with the Expectation WOMAC (Western Ontario and Mc Master Universities Osteoarthritis index). The Expectation WOMAC was adapted from the original version of the WOMAC [36]. The WOMAC is a 24-item index including three subscales: pain (five items), stiffness (two items) and functional disability (17 items). With excellent validity and reliability, it has already been successfully translated into Chinese [37, 38]. The Expectation WOMAC consisted of accurately the same domains of the WOMAC. Since the goal was to assess the patient’s expectation after surgery, we made a slight modification to the initial wording of the questions: we employed the question “How do you expect to feel six months after the arthroplasty?” rather than “How much pain or stiffness and how many limitations are you experiencing currently?” A five-point Likert scale was adopted the same as in the original WOMAC. Every single item was counted on a scale of 0 (extreme) - 4 (none). The total score ranged from 0 to 96. The higher the score is, the higher the expectations are.

### Study design

In the orthopedics outpatient department of our hospital, participants were asked to complete the SC-THRES/TKRES and the Expectation WOMAC within 10 min, respectively. Demographic variables were collected. Two weeks later, prior to surgery, they were asked to complete the surveys for the second time. Patients were excluded if they had experienced any new treatments since the completion of the first interview.

### Structural validity and reliability

The dimensionality of the original English version has been determined [25, 27]. In this study, the Confirmatory Factor Analysis (CFA) was used to test a priori construct of several dimensions identical to the original English version. It was suggested that the values of the two indexes, the comparative fit index (CFI) and goodness-of-fit index (GFI), greater than roughly 0.90 may indicate reasonably good fit of the model [39]. Furthermore, the root mean square error of approximation (RMSEA) was considered as a measure of fit. The criterion of judgment was as follows: RMSEA≤0.05, close approximate fit; RMSEA between 0.05 and 0.08, reasonable error of approximation; RMSEA≥0.10, poor fit.

The internal consistency and test-retest reliability were used to evaluate the reliability. The Cronbach’s alpha coefficient (α) was used to assess the internal consistency, where a value greater than 0.7 was considered adequate [33]. The test-retest reliability was measured by intraclass correlation coefficient (ICC, two-way random effects model, absolute agreement). The values of ICC were reported with 95% confidence internals (CIs). When ICC was above 0.80, the result was considered as good reproducibility [40]. To determine agreement, the Bland-Altman plots were made, in which the mean difference (d) between the first and second measurements with corresponding 95% CI and the 95% Limits Of Agreement (LOA) were presented (d ± t_n-1_× SD_d_) [41]. A Bland-Altman plot not only described the mean score of the two measurements and the difference between them, but also was used to assess whether there was a systematic bias between the test and retest of the SC-THRES/TKRES.

### Construct validity

Good convergent validity indicates good correlations between the questionnaire and other measures of similar concepts. Convergent validity was obtained by calculating the Pearson’s correlation coefficients (*r*) between the total scores of the SC-THRES/TKRES and the Expectation WOMAC. The criterion of judgment was as follows: *r* = 0–0.20, poor; *r* = 0.21–0.40, fair; *r* = 0.41–0.60, moderate; *r* = 0.61–0.80, good; *r* = 0.81–1.0, excellent [42]. Convergent construct validity of SC-THRES/TKRES was tested using a hypothesis. We hypothesized the Expectation WOMAC would have moderate to strong correlations (0.40–0.80) with the SC-THRES/TKRES. Moreover, Bland-Altman analyses were performed to determine whether bias occurred.

### Floor and ceiling effects

We conducted this analysis by calculating the percentage frequency of the highest or lowest score achieved by subjects, which a value higher than 15% was considered to be significant.

### Statistical analysis

Statistical analysis was performed by using SPSS version 19.0 (SPSS, Chicago, USA) and the Medcalc software version 16.4 (Medcalc, Ostend, BEL). Means and standard deviation (SD) were applied for the patient characteristics and scores of the questionnaires. *P* values less than 0.05 were considered statistically significant.

## Results

### Cross-cultural adaption

As a result of the distance measure of “one block” is not popular in China, so is “mile”, the expert committees discussed and decided to use “100 m” instead if “one block”, “1500 m” instead of “one mile”. According to the situation in China, the expert committee specified the “exercise or participate in sports” with “brisk walking or jogging” in our study.

### Sociodemographic and clinical characteristics

In total, 270 patients were recruited into the registry. A total of 14 patients (5.2%) who met the exclusion criteria were not included in the analysis. Finally, a total of 256 eligible patients (104 hip OA, 51 AS, 101 knee OA) were enrolled in the research. Females of hip OA group, Knee OA group and AS group numbered 55 (52.4%), 68 (67.3%) and 5 (9.8%), respectively. All the patients completed the questionnaires without any difficulties. Of the 256 patients, 202 patients (86 hip OA, 42 AS, 74 knee OA) were included in the test–retest reliability. More clinical characteristics and the distribution of the scores were shown in Table 1.

**Table 1:** Patient characteristics and mean total scores of the questionnaires

Characteristics^a^	OA		AS
	Hip	Knee	
Number	104	101	51
Gender			
female	55 (52.4)	68 (67.3)	5 (9.8)
Age in years	63.8 ± 9.4	65.1 ± 8.4	41.3 ± 12.1
Body mass index in kg/m^2^	24.9 ± 3.1	26 ± 2.2	21.7 ± 2.0
Education			
Low	63 (60.6)	65 (64.4)	18 (35.3)
Medium	29 (27.9)	26 (25.7)	27 (52.9)
High	12 (11.5)	10 (9.9)	6 (11.8)
Living situation			
Living alone	20 (19.2)	24 (23.1)	8 (15.7)
Living with partner and/or children	84 (80.8)	77 (76.2)	43 (84.3)
Expectation Score A	75.3 ± 12.7	72.6 ± 11.1	79.5 ± 12.9
Expectation Score B	73.9 ± 9.6	71.9 ± 8.3	78.4 ± 11.3
Expectation WOMAC Score	88.1 ± 9.9	84.9 ± 10.8	91.1 ± 9.6

*OA* osteoarthritis, *AS* ankylosing spondylitis, *WOMAC* Western Ontario and Mc Master Universities Osteoarthritis index

^a^Quantitative variables: mean standard deviation; categorical variables: frequency (percentage)

### Floor and ceiling effects

The lowest score of HSS-THRES/TKRES was not observed in any participant. While the highest score was observed in two AS patients (3.92%).

### Simplified Chinese hip replacement expectations survey

#### Structural validity

In this present study, the CFA was used to test the structural validity. The results of CFA revealed good fit (χ^2^ = 272.35, CFI = 0.933, GFI = 0.915, RMSEA = 0.052, more details were shown in Additional file 2).

#### Reliability

**Fig. 1:**
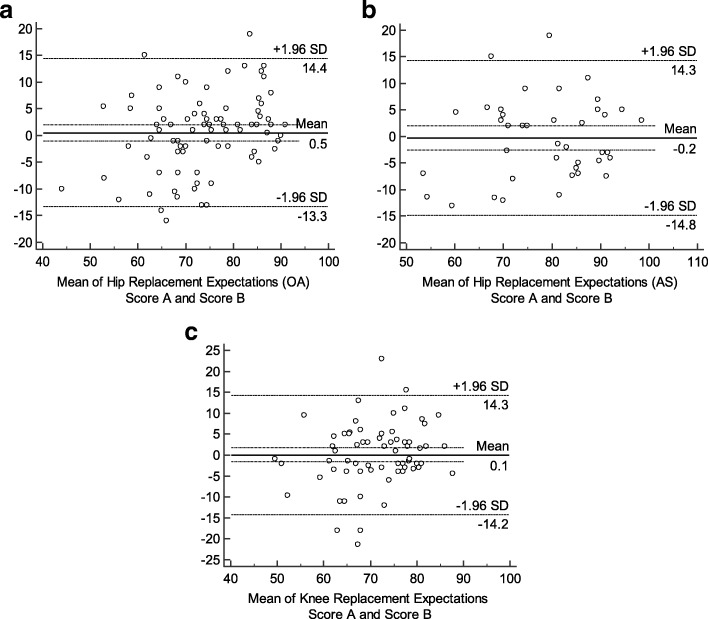
Bland–Altman plots of test–retest reliability of the SC-THRES/TKRES. The plots are for the (**a**) hip OA group, (**b**) AS group and (**c**) knee OA group. Each data point indicates how the difference between the two test sessions for an individual patient compares to the mean of the two sessions for scores of the SC-THRES/TKRES. The long dashed line shows the 95% (±1.96 SD) limits of agreement. SD, standard deviation; SC-THRES, simplified Chinese total hip replacement expectations survey; SC-TKRES, simplified Chinese total knee replacement expectations survey

The SC-THRES showed good test-retest reliability, both in hip OA and AS groups. In hip OA group, the mean score of the retest (73.9 ± 9.6) was similar to the first test (75.3 ± 12.7). The ICC between the two sessions was 0.878 (95% CI, 0.818–0.920). In AS group, the mean score of the second test (78.4 ± 11.3) was similar to the former result (79.5 ± 12.9). ICC for the test-retest was 0.893 (95% CI, 0.801–0.942). The internal consistency was good. The Cronbach’s α values were 0.814 and 0.815 for hip OA group and AS group, respectively (Table 2). As for the agreement, the Bland-Altman plots of the two groups showed that zero lies within the 95% CI of the mean difference (d) between the first and second measurement of the SC-THRES, indicating no bias (Fig. 1a, b). The 95% LOA were 0.54 ± 13.85 (hip OA group) and − 0.23 ± 14.53 (AS group).

**Table 2:** Internal consistency and reliability of the SC-THRES/TKRES

Scale	Cronbach’s α coefficient	ICC (95% CIs)
Hip Replacement Expectations Score		
OA group	0.814	0.878 (0.818–0.920)
AS group	0.815	0.893 (0.801–0.942)
Knee Replacement Expectations Score	0.808	0.806 (0.693–0.878)

*SC-THRES* Simplified Chinese Total Hip Replacement Expectations Survey, *SC-TKRES* Simplified Chinese Total Knee Replacement Expectations Survey, *ICC* intraclass correlation coefficient, *CIs* confidence internals, *OA* osteoarthritis, *AS* ankylosing spondylitis

#### Validity

**Fig. 2:**
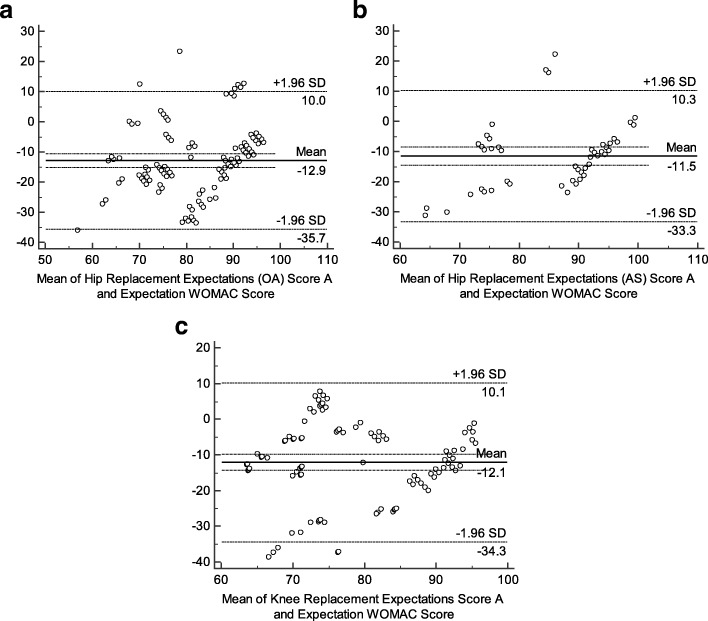
Bland–Altman plots of concurrent validity of the SC-THRES/TKRES. The plots are for the (**a**) hip OA group, (**b**) AS group and (**c**) knee OA group. Each data point indicates how the difference between the two test sessions for an individual patient compares to the mean of the two sessions for scores of the SC-THRES/TKRES. The long dashed line shows the 95% (±1.96 SD) limits of agreement. SD, standard deviation; SC-THRES, simplified Chinese total hip replacement expectations survey; SC-TKRES, simplified Chinese total knee replacement expectations survey

Pearson’s coefficients were shown in Table 3. The result demonstrated that the correlations between the SC-THRES and the Expectation WOMAC (0.490, *p* < 0.01; 0.541, *p* < 0.01) were moderate for hip OA group and AS group, respectively. This was in line with our hypotheses. The Bland-Altman plots (Fig. 2a, b) showed that the 95% LOA were − 12.85 ± 22.85 (hip OA group) and − 11.51 ± 21.83 (AS group). The mean SC-THRES score of hip OA group was 12.72 points lower than the mean Expectation WOMAC score. And the mean SC-THRES score of AS group was 11.51 points lower than that of the Expectation WOMAC. Systematic bias could be found in the two groups since zero was not in the 95% CI of mean difference (d).

**Table 3:** Construct validity^a^ of the SC-THRES/TKRES

	expectation WOMAC score
Hip Replacement Expectations Score	
OA	0.490 (*P* < 0.01)
AS	0.541 (*P* < 0.01)
Knee Replacement Expectations Score	0.465 (*P* < 0.01)

*SC-THRES* Simplified Chinese Total Hip Replacement Expectations Survey, *SC-TKRES* Simplified Chinese Total Knee Replacement Expectations Survey, *OA* osteoarthritis, *AS* ankylosing spondylitis, *WOMAC* Western Ontario and Mc Master Universities Osteoarthritis index

^a^Calculated by Pearson’s correlation of the SC-THRES/TKRES with expectation WOMAC score in different groups

### Comparison between hip OA group and AS group

**Fig. 3:**
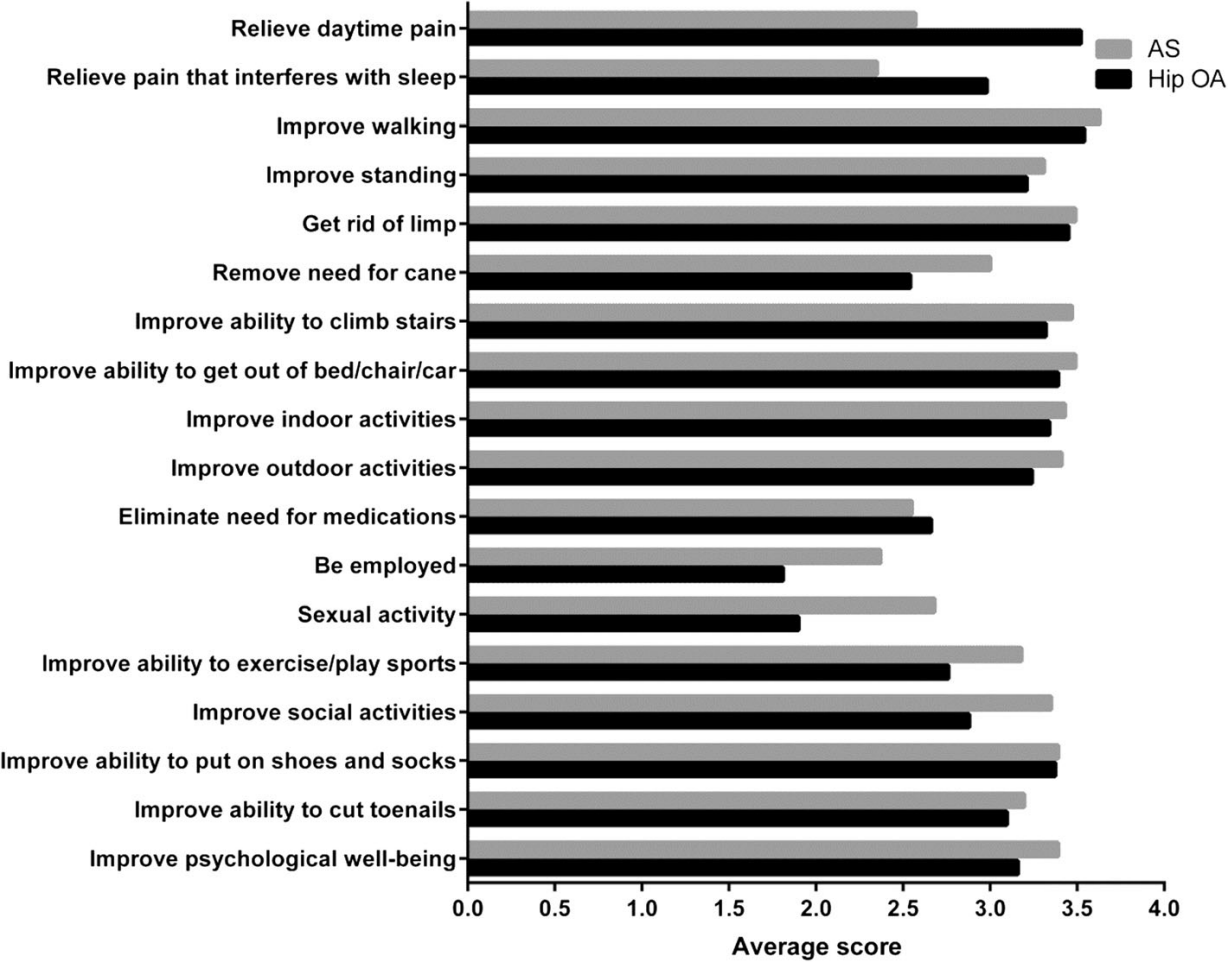
Total hip replacement patients’ responses to SC-THRES. OA, osteoarthritis; AS, ankylosing spondylitis; SC-THRES, simplified Chinese total hip replacement expectations survey

Average scores for each item of the SC-THRES were presented in Fig. 3. The most important expectation cited by patients was item 3 (improving walking), both in hip OA and AS group. The least important expectations cited were item 12 (be employed for monetary reimbursement) in hip OA group and item 2 (relieve pain that interferences with sleep) in AS group. Compared to hip OA group, the mean score of AS group for every individual item was mostly higher except for item 1 (relieve daytime pain), 2 (relieve pain that interferences with sleep) and 11 (eliminate the need for medications). There were significant differences between hip OA group and AS group in item 1 (relieve daytime pain, hip OA: 3.519, AS: 2.569; *p* = 0.009), 6 (remove need for cane, hip OA: 2.538, AS: 3; *p* = 0.018), 12 (be employed for monetary reimbursement, hip OA: 1.808, AS: 3.373; *p*<0.001), 13 (sexual activity, hip OA: 1.904, AS: 2.686; *p* = 0.012), 14 (improve ability to exercise/play sports, hip OA: 2.760, AS: 3.176; *p* = 0.008) and 15 (improve social activities, hip OA: 2.885, AS: 3.353; *p* = 0.004).

### Simplified Chinese knee replacement expectations survey

#### Structural validity

Also the results of CFA demonstrated good fit (χ^2^ = 302.10, CFI = 0.929, GFI = 0.927, RMSEA = 0.054, more details were shown in Additional file 2).

#### Reliability

The mean score of the retest was 71.9 ± 8.3, which was similar to the first result (72.6 ± 11.1). The ICC between the first and second assessment was 0.806 (95% CI 0.693–0.878), which indicated good test-retest reliability. The internal consistency result was good as well. The Cronbach’α was 0.808 for the overall SC-TKRES (Table 2). In addition, the Bland-Altman plot (Fig. 1c) showed no systematic bias, in which zero lies within the 95% CI of the mean difference (d) between the measurements of the SC-TKRES in the two sessions. The 95% LOA were 0.05 ± 14.24.

#### Validity

As shown in Table 3, the result indicated good convergent validity. The correlation between the SC-TKRES score (first assessment) and the Expectation WOMAC total score (0.465, *p*<0.01) was moderate, which conformed to the hypotheses. The Bland-Altman plot showed the 95% LOA were − 12.09 ± 22.24 (Fig. 2c). Total score of the Expectation WOMAC was 12.09 points higher than that of the SC-TKRES and different from zero significantly, which indicated systematic bias.

## Discussion

OA is a highly prevalent disease in the older population [43]. Although the prevalence of AS is lower than OA, the amount of patients with AS is also considerable because of the huge population of China, which lead to a heavy burden. TJA is an effective way in reducing pain and improving function in patients with end-stage OA and AS. As the demand for TJA continues to grow, understanding the factors associated with the outcomes of this procedure has become even more important. Nowadays clinical surgeons are placing more emphasis on self-reported outcome assessment. But patients’ preoperative expectations have seldom been studied. Analyzing patients’ expectations may help surgeons to understand better what is important for their patients and therefore enhance patients’ satisfaction and the doctor–patient relationship. In addition, the differences of age and state between AS and OA may result in discrepancies of expectation, of which may be unrealistic and need to be educated before surgery. The HSS-THRES and the HSS-TKRES are widely used for patients undergoing TJA, but to our knowledge, these widely used tools have not been validated in a Chinese population. So it is greatly meaningful to translate the questionnaires into Chinese. The translation process was nicely developed. All patients completed the questionnaires without any difficulties, which meant the translated questionnaires had good cultural acceptability. No significant floor and ceiling effects were observed in our study.

The interval time was two weeks between the first and second test in our study. It was appropriate to assess the test-retest reliability for the reason that the interval was long enough to keep patients from completing the questionnaires from memory, and short enough to prevent dramatic changes in expectations. According to the results, both simplified Chinese surveys showed good test-retest reliability. The ICCs for the total scores (0.878, 0.893 and 0.806 for hip OA group, AS group and knee OA group, respectively) were all higher than the minimum criterion of 0.80 recommended by Fleiss et al. [40], similar to the results of validated Dutch [28] and French [29] versions. As a result of no available information regarding the reliability of the original tool, we did not compare our results with the English version. In addition, no bias between measurements of the two sessions was observed in the Bland-Altman analysis, which was in accordance with other cross-cultural adapted studies [28, 29]. Determined by Cronbach’s α, the internal consistency of the simplified Chinese surveys were all good (0.814, 0.815 and 0.808 for hip OA group, AS group and knee OA group, respectively), which was also conformed with the criterion suggested by Terwee et al. [33]. With respect to surveys applied in patients with hip OA, the Cronbach’s α (0.814) was above the original English version (0.77) [27] and the French version (0.72) [29], but slightly lower than the Dutch version (0.86) [28]. As for the knee OA group, present value (0.808) was in line with the original version (0.79) [27] and the French version (0.82) [29], but lower than the Dutch version (0.91) [28].

Construct validity was demonstrated by assessing the correlation between the SC-THRES/TKRES scores and Expectation WOMAC scores. Until now, no so-called “gold standard” can be used to optimally evaluate patients’ expectations. We employed the Expectation WOMAC for the reason that (1) it had been first presented and demonstrated by Haddad et al. [26] and had been used in other studies [28, 29]; (2) the Chinese WOMAC is proved to be reliable and valid [37, 38]. And we made a slight modification to result in the Expectation WOMAC. On the basis of the criterion, the Pearson’s correlations between the SC-THRES/TKRES and Expectation WOMAC were moderate (0.49 for hip OA, 0.541 for hip AS and 0.465 for knee OA). Despite this moderate result, we found a systematic bias in the Bland-Altman analysis. Compared to the SC-THRES/TKRES, the mean scores of the Expectation WOMAC were higher. However, the same trend was found in the Dutch and French version [28, 29]. Van et al. [28] thought that the way the Expectation WOMAC was adapted from the original WOMAC resulted in answers whereby the patients also considered the current status. However, Neuprez et al. [29] believed that the response’s categories and codification of the two measures were different. Our view was different from theirs. The subscales of Expectation WOMAC included pain, stiffness and functional disability. These were the main symptoms which patients wanted to get rid of as soon as possible. In addition to the items related to these symptoms, the THRES/TKRES also included psychological well-being, sexual activity and employment, which were not that important for patients. These sections may lower the mean scores of the THRES/TKRES.

The mean total score of AS group was higher both in the Expectation WOMAC and the SC-THRES. The majority of the mean score for an individual item revealed the same trend. It was suggested that item was of some importance to patients when the average score was ≥2 points. The majority of the average scores were over 2.5 and some were even over 3, indicating they were important to patients. Patients with AS undergoing THA were of worse functional status and had higher expectations than those with hip OA [44]. Not only hip OA patients, but also AS patients showed the highest expectations to improve walking. This was because patients in both groups were almost end-staged with severe pain and dysfunction in the hips, which limited their abilities to walk. Patients with hip OA showed higher expectations for pain relief during daytime than those with AS. This can be explained because many patients in AS group were bony ankylosis with less pain currently. The mean age of hip OA group and AS group were 63.8 ± 9.4 and 41.3 ± 12.1, respectively. The majority of patients in hip OA group were retired from work and they showed lower expectations to be employed. However, patients with AS were always younger, and they were unable to work because of their physical disabilities. They were expected to be endorsed by family and society through rehabilitation. Similarly, patients with AS had more expectations in sexual activity than those with hip OA.

There were two strengths of our study. First, all the patients completed the questionnaires in the outpatient room of our hospital to avoid missing values. Second, to our knowledge, it was the first time that the surveys were validated in patients with AS.

With no doubt, there were some limitations in the current study that should be considered. First, considering the absence of validated tools assessing preoperative expectations, studying such questionnaire remains difficult. Although the Chinese WOMAC is reliable and valid, the psychometric properties of the Chinese version Expectation WOMAC are unknown. Without a doubt, this may result in some issues. For example, patients may take this adapted WOMAC as the original and consider the current state. Taking the expectations of orthopedic surgeons as reference would be an alternative way [28]. However, this is doubtful owing to the discrepancies between the expectations of orthopedic surgeons and those of patients [19, 20]. Thus we took the closest tool as a reference to evaluate the validity in our study. Second, the sample was limited in size, which may not be representative of the whole Chinese population. It was noteworthy that the sample of AS was too small for assessing the structural validity, we conducted this assessment among the OA and AS sample together. Last but not least, we translated the surveys into Mandarin, the official language of China, but traditional Chinese languages are still widely used in several areas of China. So it will be necessary to translate the surveys into traditional Chinese languages in the future.

## Conclusion

In summary, the HSS-THRES and HSS-TKRES have been nicely adapted into Chinese versions with good psychometric properties. As self-reported questionnaires, the SC-THRES and SC-TKRES are reliable and valid for a Chinese population with OA or AS undergoing TJAs. Therefore, we suggest that the two questionnaires can be used to investigate patients’ expectations before surgeries. And patients’ expectations should be part of preoperative assessments. Future studies would be conducted to assess the content validity, cross-cultural validity and responsiveness of these two questionnaires.

## Additional files

Additional file 1: **Table S1.** Characteristics of patients in the pre-testing process. (DOCX 19 kb)

Additional file 2: **Table S2.** Results of CFA for each dimension of SC-THRES. (DOCX 17 kb)

## Abbreviations

AS: Ankylosing spondylitis
CFA: Confirmatory Factor Analysis
CFI: Comparative fit index
CIs: Confidence internals
GFI: Goodness-of-fit index
HSS-THRES: Hospital for Special Surgery Total Hip Replacement Expectations Survey
HSS-TKRES: Hospital for Special Surgery Total Knee Replacement Expectations Survey
ICC: Intraclass correlation coefficient
LOA: Limits Of Agreement
OA: Osteoarthritis
RMSEA: Root mean square error of approximation
SC-THRES: Simplified Chinese Total Hip Replacement Expectations Survey
SC-TKRES: Simplified Chinese Total Knee Replacement Expectations Survey
SD: Standard deviation
THA: Total hip arthroplasty
TJAs: Total joint arthroplasties
TKA: Total knee arthroplasty
WOMAC: Western Ontario and Mc Master Universities Osteoarthritis index
