# Supplementary material for: Simplified Chinese version of hip and knee replacement expectations surveys in patients with osteoarthritis and ankylosing spondylitis: cross-cultural adaptation, validation and reliability
Source: BMC Musculoskelet Disord. 2018 Jul 21;19:247. doi: 10.1186/s12891-018-2129-0 (PMC6054857; doi:10.1186/s12891-018-2129-0)
Supplement: Supplementary file 1 — Table S1. Characteristics of patients in the pre-testing process. (DOCX 19 kb) [file 12891_2018_2129_MOESM1_ESM.docx]

**Additional Table** **S1**

Characteristics of patients in the pre-testing process.

| Characteristics^a^ | OA | | AS |
| --- | --- | --- | --- |
|  | Hip | Knee |  |
| Number | 7 | 9 | 4 |
| Gender |  |  |  |
| Female | 4 (57.1) | 6 (66.7) | 0 |
| Age in years | 65.9 ± 6.2 | 63.6 ± 7.2 | 39.3 ± 11.3 |
| Body mass index in kg/m^2^ | 24.1 ± 2.5 | 25.8 ± 2.2 | 20.3 ± 1.0 |
| Education |  |  |  |
| Low | 5 (71.4) | 5 (55.6) | 1 (25.0) |
| Medium | 2 (28.6) | 3 (33.3) | 2 (50.0) |
| High | 0 | 1 (11.1) | 1 (25.0) |
| Living situation |  |  |  |
| Living alone | 2 (28.6) | 2 (22.2) | 1 (25.0) |
| Living with partner and/or children | 5 (71.4) | 7 (77.8) | 3 (75.0) |

**OA: osteoarthritis; AS: ankylosing spondylitis; WOMAC: Western Ontario and Mc Master Universities Osteoarthritis index**

**^a^ Quantitative variables: mean standard deviation; categorical variables: frequency (percentage)**
