# Supplementary material for: Simplified Chinese version of hip and knee replacement expectations surveys in patients with osteoarthritis and ankylosing spondylitis: cross-cultural adaptation, validation and reliability
Source: BMC Musculoskelet Disord. 2018 Jul 21;19:247. doi: 10.1186/s12891-018-2129-0 (PMC6054857; doi:10.1186/s12891-018-2129-0)
Supplement: Supplementary file 2 — Table S2. Results of CFA for each dimension of SC-THRES. (DOCX 17 kb) [file 12891_2018_2129_MOESM2_ESM.docx]

**Additional Table** **S2**

Results of CFA for each dimension of SC-THRES.

|  | RMSEA | GFI | CFI |  |
| --- | --- | --- | --- | --- |
| Pain | 0.053 | 0.921 | 0.951 |  |
| Walking | 0.065 | 0.945 | 0.954 |  |
| Necessary activities | 0.050 | 0.930 | 0.923 |  |
| Non-necessary activities | 0.049 | 0.954 | 0.938 |  |

**CFA: Confirmatory Factor Analysis; SC-THRES: Simplified Chinese Total Hip Replacement Expectations Survey; RMSEA: Root Mean Square Error of Approximation; GFI: Goodness-of-Fit Index; CFI: Comparative Fit Index**

**Additional Table** **S3**

Results of CFA for each dimension of SC-TKRES.

|  | RMSEA | GFI | CFI |  |
| --- | --- | --- | --- | --- |
| Pain | 0.053 | 0.950 | 0.936 |  |
| Walking | 0.049 | 0.948 | 0.941 |  |
| Necessary activities | 0.056 | 0.937 | 0.936 |  |
| Non-necessary activities | 0.050 | 0.926 | 0.921 |  |

**CFA: Confirmatory Factor Analysis; SC-TKRES: Simplified Chinese Total Knee Replacement Expectations Survey; RMSEA: Root Mean Square Error of Approximation; GFI: Goodness-of-Fit Index; CFI: Comparative Fit Index**
